# Convergent reductive evolution in bee-associated lactic acid bacteria

**DOI:** 10.1128/aem.01257-24

**Published:** 2024-10-23

**Authors:** Ana Pontes, Marie-Claire Harrison, Antonis Rokas, Carla Gonçalves

**Affiliations:** 1 Associate Laboratory i4HB, Institute for Health and Bioeconomy and UCIBIO, Applied Molecular Biosciences Unit, Department of Life Sciences, NOVA School of Science and Technology, Universidade NOVA de Lisboa, Caparica, Portugal; 2 UCIBIO-i4HB, Departamento de Ciências da Vida, Faculdade de Ciências e Tecnologia, Universidade Nova de Lisboa, Caparica, Portugal; 3 Department of Biological Sciences, Vanderbilt University, Nashville, Tennessee, USA; 4 Evolutionary Studies Initiative, Vanderbilt University, Nashville, Tennessee, USA; University of Nebraska-Lincoln, Lincoln, Nebraska, USA

**Keywords:** convergent evolution, reductive evolution, fructophilic lactic acid bacteria (FLAB), bee symbionts, machine learning, gene loss

## Abstract

Distantly related organisms may evolve similar traits when exposed to similar environments or engaging in certain lifestyles. Several members of the Lactobacillaceae [lactic acid bacteria (LAB)] family are frequently isolated from the floral niche, mostly from bees and flowers. In some floral LAB species (henceforth referred to as bee-associated LAB), distinctive genomic (e.g., genome reduction) and phenotypic (e.g., preference for fructose over glucose or fructophily) features were recently documented. These features are found across distantly related species, raising the hypothesis that specific genomic and phenotypic traits evolved convergently during adaptation to the floral environment. To test this hypothesis, we examined representative genomes of 369 species of bee-associated and non-bee-associated LAB. Phylogenomic analysis unveiled seven independent ecological shifts toward the bee environment in LAB. In these species, we observed significant reductions of genome size, gene repertoire, and GC content. Using machine leaning, we could distinguish bee-associated from non-bee-associated species with 94% accuracy, based on the absence of genes involved in metabolism, osmotic stress, or DNA repair. Moreover, we found that the most important genes for the machine learning classifier were seemingly lost, independently, in multiple bee-associated lineages. One of these genes, acetaldehyde–alcohol dehydrogenase (*adhE*), encodes a bifunctional aldehyde–alcohol dehydrogenase which has been associated with the evolution of fructophily, a rare phenotypic trait that is pervasive across bee-associated LAB species. These results suggest that the independent evolution of distinctive phenotypes in bee-associated LAB has been largely driven by independent losses of the same sets of genes.

**IMPORTANCE:**

Several LAB species are intimately associated with bees and exhibit unique biochemical properties with potential for food applications and honeybee health. Using a machine learning-based approach, our study shows that adaptation of LAB to the bee environment was accompanied by a distinctive genomic trajectory deeply shaped by gene loss. Several of these gene losses occurred independently in distantly related species and are linked to some of their unique biotechnologically relevant traits, such as the preference for fructose over glucose (fructophily). This study underscores the potential of machine learning in identifying fingerprints of adaptation and detecting instances of convergent evolution. Furthermore, it sheds light onto the genomic and phenotypic particularities of bee-associated bacteria, thereby deepening the understanding of their positive impact on honeybee health.

## INTRODUCTION

Ecological lifestyles shape the content of microbial genomes. For instance, microorganisms that engage in strictly dependent relationships with their hosts, such as parasites or intracellular symbionts, generally undergo major reduction in genome size and gene repertoire (1, 2). Numerous examples have been found across microorganisms such as fungi and bacteria. For instance, species belonging to the fungal lineage Microsporidia, strictly composed of obligate intracellular parasites, are devoid of many central pathways involving carbohydrate and amino acid metabolism, exhibiting some of the most compact eukaryotic genomes (3–5). In bacteria, genome reduction-associated symbiotic lifestyles have been identified in independent lineages from diverse bacterial groups (6, 7). In addition to reduced gene repertoires, symbiotic bacterial genomes sometimes exhibit other distinctive features such as rapid sequence evolution, codon reassignments, or low GC content (6). While most losses are possibly the result of relaxed selection acting on functions that can be supplied by the host such as those involved in metabolic processes, others might be the result of genetic drift during the shift to a more confined and restricted habitat, where the effect of small effective population results in reduced opportunities for gene exchange and homologous recombination, originating strong intergenerational bottlenecks (1, 6, 8–10).

However, other gene losses can be adaptive when organisms are adjusting to a new environment (11). For instance, loss of function mutations in genes involved in multiple pathways (e.g., DNA repair or adhesion to neutrophils) may increase pathogenicity or drug resistance in bacterial species, providing a selective advantage for survival in the human body (12).

Recently, a unique group of lactic acid bacteria (LAB) belonging to *Fructobacillus* and *Lactobacillus* genera has been described based on their unusual metabolic characteristics and their distinctive ecological association with fructose-rich environments involving flowers, fruits, and flower-visiting insects, such as bees (13–15). Many of these bacteria have been described as bee symbionts (16, 17). However, they are found across the floral niche, suggesting that they inhabit the bee gut but can thrive outside their hosts. Adaptation to this fructose-rich environment is thought to have promoted a major metabolic remodeling toward efficient fructose utilization because these species use fructose more efficiently than glucose (15, 18). This unusual metabolic feature, dubbed as fructophily, is seemingly associated with the partial or complete loss of a gene encoding a bifunctional acetaldehyde–alcohol dehydrogenase (*adhE*), rendering these species unable to engage in alcoholic fermentation (18–20). Many fructophilic lactic acid bacteria (FLAB) present reduced gene repertoires associated with multiple functions such as carbohydrate metabolism (e.g., phosphotransferase system) (16). Whether these losses result from adaptation to the floral environment, from the symbiotic relationship with bees, or both remains elusive.

Hence, we set out to look for evidence of convergent evolution in floral bacteria (henceforth referred to as bee associated) across the entire Lactobacillaceae family (369 species, henceforth referred to as LAB), which includes four distinct clades of bee-associated species (21, 22). We used a phylogenomic framework onto which ecological and genomic information was mapped. Supporting previous reports, we observed that genome reduction was pervasive across bee-associated LAB. Specifically, we observed that absence of *adhE* was a distinctive feature of bee-associated LAB. We found additional genomic fingerprints of convergent evolution by employing a machine learning algorithm, which showed that association with the bee environment can be predicted from functional genomic data with very high (94%) accuracy. While the *adhE* gene was the most important predictor, accuracy remained unaltered when we removed *adhE* from the data set. These features included genes involved in carbohydrate and amino acid metabolism, osmotic stress, and DNA repair, which are distinctively absent from bee-associated LAB. Moreover, we found that some of these genes were likely lost in the branches leading to distantly related bee-associated clades. These results revealed that convergent adaptation to the bee environment in LAB was seemingly accomplished through highly similar evolutionary mechanisms, some of which (i.e., loss of *adhE*) are associated with distinctive metabolic features (i.e., fructophily).

## RESULTS

### Multiple instances of ecological association with the bee environment across LAB

**Fig 1:**
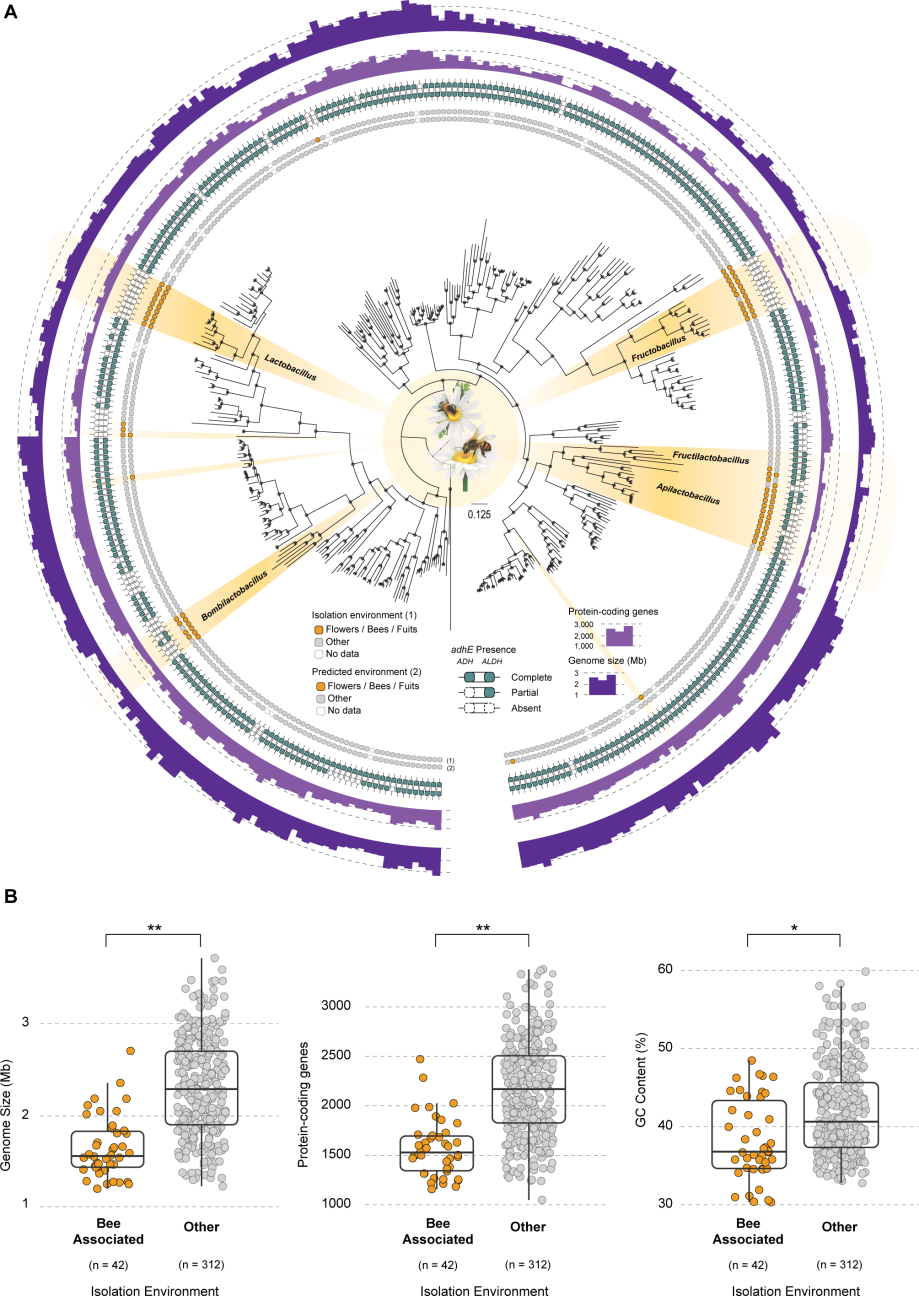
Phylogenomic tree of the Lactobacillaceae. (**A**) Maximum likelihood phylogenomic tree comprising 369 LAB species inferred from the concatenated alignment of 180 single-copy orthogroups and rooted with *Lactococcus lactis* and *Enterococcus massiliensis*. The isolation source and predicted environment are depicted in the first two rings, respectively. The species isolated from bee-associated environments are highlighted in yellow. Presence/absence of *adhE*, number of protein-coding genes, and genome size are also shown in the other three rings. (**B**) Boxplots depicting comparisons between bee-associated species and others regarding genome size, number of protein-coding genes, and GC content. The respective statistical significance after phylogenetically informed phylANOVA is displayed for each feature. *P < 0.05, **P < 0.01.

To ascertain the distribution of bee association across lactic acid bacteria, we retrieved all representative genomes from species belonging to the Lactobacillaceae (LAB) family (369, as of 19 January 2023) (Table S1) and inferred a phylogenomic tree using 180 single-copy orthogroups (SCOs). The resulting species tree (Fig. 1; Fig. S1) recapitulates the phylogenetic relationships among the main lineages within LAB (21).

We next inspected ecological association with the floral environment (bees, flower, and fruits; henceforth referred to as the bee environment) across the entire data set. Ecological association was established according to the substrate of isolation of the strain for which the genome sequence was obtained. Most of the 369 species were isolated from anthropic environments (processed foods) and the gut of mammals (Table S1); however, 42 species were isolated from bee-associated environments. We identified four distinct instances of bee association involving entire clades (Fig. 1; Fig. S1): *Bombilactobacillus*, *Fructobacillus*, *Fructilactobacillus*–*Apilactobacillus* genera, and a subclade within the *Lactobacillus* genus; and three instances of floral association in distantly related single species: *Companilactobacillus musae*, *Holzapfelia floricola*, and *Secundilactobacillus yichangensis*. Most species (~60%) were isolated from bees, while the remaining 40% were isolated from flowers, fruits, or honey (Table S1).

Using this classification, we can infer that seven independent ecological shifts toward bee-associated environments likely occurred within LAB. The clades identified include species described in previous studies as being FLAB (15, 21).

### Genome reduction is pervasive across bee-associated LAB

Distinctive genome reduction was previously reported for several FLAB isolated from the floral niche (21). We therefore assessed the genome sizes of 369 species belonging to LAB. We observed that genome size considerably varies across the family (1.2–3.7 Mb). When comparing bee-associated species with all the others and accounting for phylogenetic relatedness (23), we observed that bee-associated genomes are significantly smaller (*P* value = 0.001, phylANOVA). In line with genome reduction, we also observed that bee-associated species encode a significantly lower number of protein-coding genes (*P* value = 0.001, phylANOVA), indicating that genome reduction is associated with gene loss (Fig. 1A and B). In addition to decreased genome size, symbiotic bacteria can also exhibit significantly lower GC content when compared to their non-symbiotic relatives (6). By analyzing the GC content across LAB, we observed that bee-associated species present significantly lower GC content than non-bee-associated species (*P* value = 0.046, phylANOVA) (Fig. 1B).

One of the most distinctive known features of bee-associated FLAB is the absence of the *adhE* (16, 21, 24). To ascertain whether the loss (total or partial) of this gene is more broadly associated with species thriving in the bee environment, we looked for the presence of AdhE across the entire data set using an hidden Markov model (HMM) based sequence similarity approach (25). We found that 80% of bee-associated species either partially (ethanol dehydrogenase domain absent) or completely lack AdhE (Fig. 1; Fig. S1). A phylogenetically informed comparison of the distributions of niche (bee and other) and AdhE (present and absent) showed that the pattern of occurrence of AdhE is statistically dependent on the niche (*P* value = 0.0008, chi-squared test).

### Machine learning analyses reveal that absence of specific genes accurately predicts bee association in LAB

We next looked for other fingerprints of adaptation to the bee environment by performing genome-wide functional annotations [Kyoto Encyclopedia of Genes and Genomes (KEGG)] for all species under study and employing a machine learning approach. Species for which a low number of proteins were mapped to a functional KEGG annotation were excluded (Fig. S2). In this way, KEGG annotations (presence/absence) and ecological information for 360 species were submitted to a trained and supervised random forest (RF) classifier (26–28).

**Fig 2:**
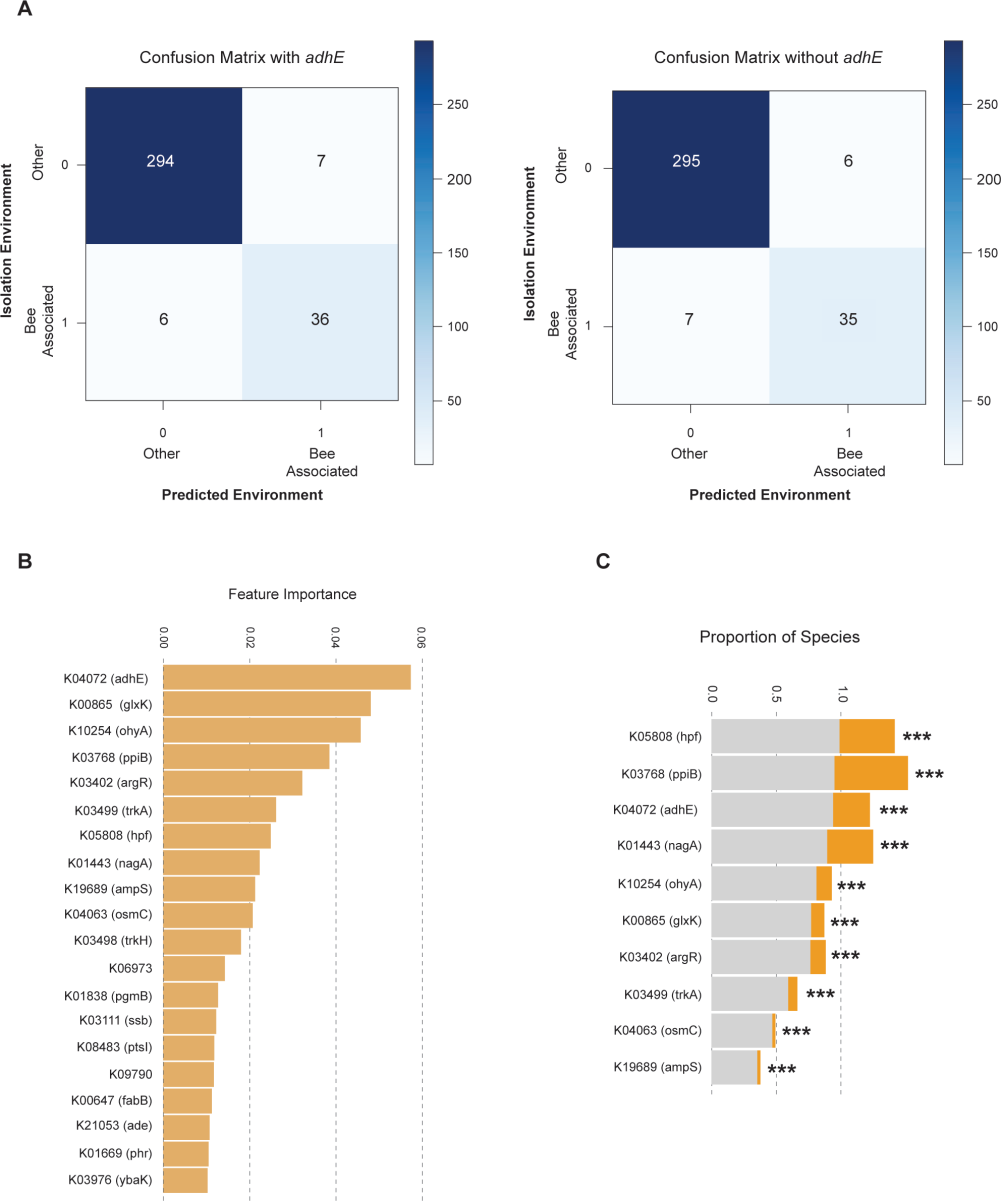
Bee-associated LAB can be predicted from genomic data with high accuracy. (**A**) Confusion matrix with and without AdhE (left and right, respectively) across 343 species of the Lactobacillaceae family. (**B**) Top 20 most important features for the RF classifier. (**C**) Distribution of the top 10 most important KEGG features across species belonging to bee-associated (orange) and non-bee-associated (gray) groups. Bar plots represent the proportion of species in which the KEGG annotation was found to be present. Statistically significant differences between the proportion of species (bee-associated and others) encoding each function are annotated next to the respective KEGG (chi-squared test). ***P < 0.001.

We obtained highly accurate predictions (balanced accuracy of 94%) of niche association (Fig. 2A). To determine which features are contributing to the RF classification, we investigated the 20 most important features (Fig. 2B). In line with the evidence of genome reduction, we found that the most important predictive genes are less prevalent in bee-associated species when compared to non-bee-associated species (Fig. 2C; *P* < 2.2e^−16^, chi-squared test). The top predictive feature was the *adhE* gene (K04072), followed by *glxK/garK* (K00865) and *ohyA* (K10254).

Since classification accuracy is being driven by absence of genes, we next confirmed that functional annotation issues did not influence our results. For that, we searched for the *ohyA* gene using an HMM-based approach across LAB proteomes and confirmed its absence in bee-associated *Fructobacillus*, *Apillactobacillus*, and in the bee-associated *Lactobacillus* subclade (Fig. S1; Table S1). Because of its importance for the RF classifier and of its notable absence in bee-associated species, we next determined its distribution across LAB species. Mapping the presence/absence of *ohyA* to the species phylogeny revealed that it follows a similar pattern to *adhE* (Fig. S1), being also statistically dependent on the niche (phylogenetically informed model testing, *P* value = 1.0398e^−07^, chi-squared test).

The other top predictive features are involved in multiple functions including carbohydrate metabolism (K01443, K08483, and K01838), amino acid metabolism (K03402), osmotic stress (K03499, K03498, and K04063), and DNA repair (K03111 and K01669) (Fig. 2B). Among these, we found *ptsI* (K08483), which was previously described to be absent in some FLAB species (16).

To test whether loss of *adhE* reduced the accuracy of RF classification, we removed the *adhE* feature from the data set and reran the RF classifier. We obtained the same accuracy and similar precision (six false negatives and seven false positives with *adhE* vs seven false negatives vs six false positives without *adhE*; Table S3).

We also looked at the species that were incorrectly classified (Table S3). Some of these may be explained by inaccuracies in our ecological categorization, which was based on the substrate of isolation of the strain for which the genome sequence was obtained. For instance, the *Apilactobacillus kunkeei* strain used in this work, which was identified as a false negative by the RF classifier (Fig. S1; Table S3), was isolated from grape wine, but most strains of this species are known to be associated and isolated from the bee environment (14, 16, 20, 22).

### Metabolism-related genes were largely lost in the most recent common ancestor of bee-associated LAB clades

To gain insight into functions that were most likely associated with the ecological shift, we next asked which genes were likely lost in the branches leading to bee-associated clades.

**Fig 3:**
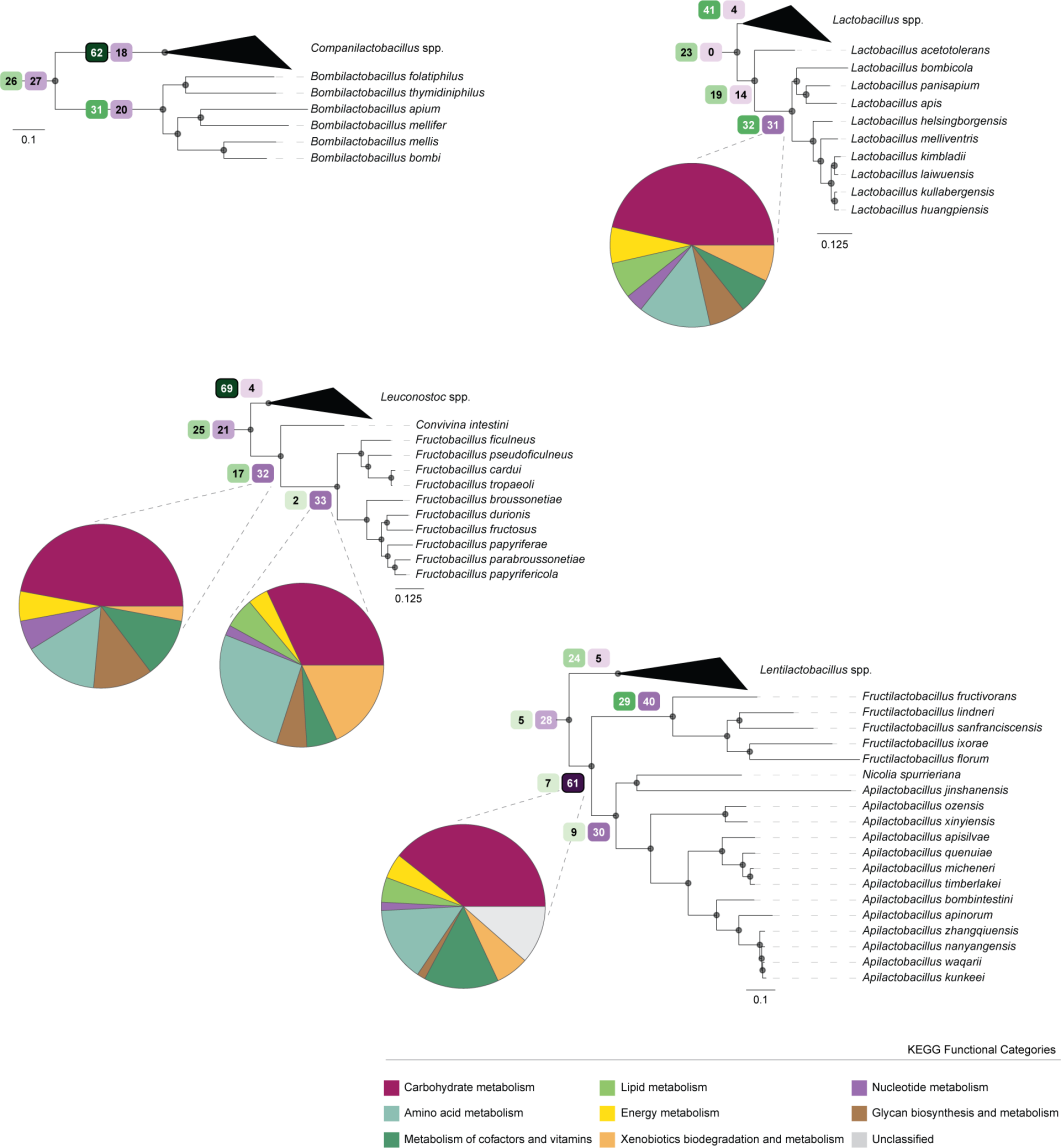
Major losses of genes related to metabolic functions that occurred in the MRCA of bee-associated clades. Pruned trees of the four main groups of bee-associated species and closest relatives showing the number of annotated KEGG functional categories that were inferred to have been gained (green tones) or lost (purple tones) in the specific nodes corresponding to the MRCA of bee-associated species. Pie charts reflect the proportion of lost KEGG functional categories (annotated in KEGG Mapper) as indicated in the key. For *Bombilactobacillus* <5 KEGG were assigned a functional category.

Hence, we looked for functions that were likely lost specifically in the MRCA of bee-associated clades and were therefore lost concomitantly with the ecological shift. This analysis revealed that significant losses took place in the MRCA of many of these clades, supporting previous evidence of genome reduction. For instance, under this model, 61 losses were inferred in the MRCA of the bee-associated *Fructilactobacillus*–*Apilactobacillus* genera (Fig. 3). Most losses across the four clades involved genes related with the metabolism of carbohydrates, amino acids, lipids, and co-factors and vitamins (Fig. 3). Importantly, we found that from the top 20 most important features for the RF classifier (Fig. 2B), 13 were inferred to have been lost in the MRCA of at least one bee-associated clade (Table S3). Five out of the 13 genes were inferred to have been lost in the MRCA of two clades. Specifically, the two most important genes for the RF classifier, *adhE* and *ohyA*, were lost in the MRCA of two bee-associated clades, independently. For instance, loss of *AdhE* was inferred in the MRCA of both *Lactobacillus* and *Fructobacillus* (Table S3). Loss of the *ptsI* gene (K08483) was also inferred in the MRCA of *Fructobacillus* and in the MRCA of *Fructilactobacillus*–*Apilactobacillus*. Two additional genes were inferred to have been independently lost in the branches leading to two distantly related bee-associated clades (K01443 and K01838). The remaining 8 out of the 13 genes were inferred to have been lost in the MRCA of one clade only. However, these genes are absent in many species belonging to other distantly related clades, suggesting that losses can occur after the ecological shift, in a stepwise manner. The remaining seven genes that are among the top 20 most important features for the random forest classifier were not found to have been lost in the MRCA of bee-associated clades, suggesting that they were probably lost at distinct time points (either before or after the ecological shift) or present a patchy distribution.

**Fig 4:**
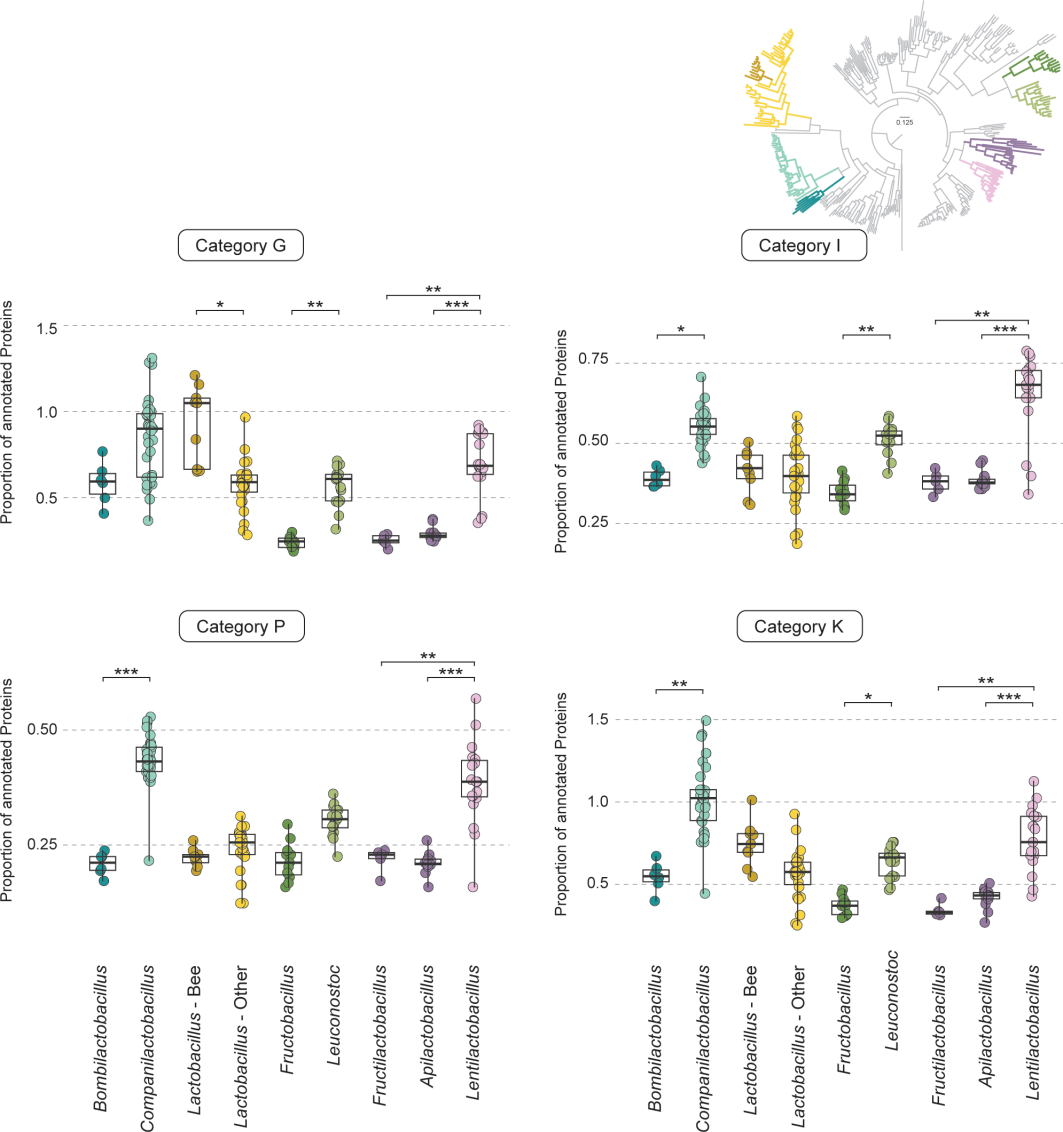
Most bee-associated clades have significantly fewer genes involved in carbohydrate and lipid transport and metabolism. Boxplots show the proportion of annotated proteins in categories G (carbohydrate transport and metabolism), I (lipid transport and metabolism), P (inorganic ion transport and metabolism), and K (transcription) for bee-associated clades and their respective sister clades. Statistically significant differences between the bee-associated groups and their closest relatives for each category were assessed using the Kruskal–Wallis test. *P < 0.05, **P < 0.01, ***P < 0.001.

We next inspected whether there was a statistically significant difference in the presence/absence pattern of certain biological functions between bee-associated species and their closest relatives. To ascertain this, we performed Cluster of Orthologous Groups (COG) analyses by comparing bee-associated species with their closest relatives (Fig. 4). For all cases, bee-associated clades had lower numbers of proteins annotated in all the categories inspected, except for *Lactobacillus* spp., for which no differences were found (Fig. S3; Table S4). For 13 out of the 26 COG categories, at least 2 bee-associated clades showed significant differences from their closest relatives. Some of the categories that showed significant differences were K (transcription), G (carbohydrate transport and metabolism), I (lipid transport and metabolism), and P (inorganic ion transport and metabolism) (Kruskal–Wallis test). Loss of carbohydrate transport and metabolism functions was previously described for species belonging to the FLAB group (16, 20, 21), and it is in line with our aforementioned results. These losses seem to be more evident in *Fructobacillus* and *Fructilactobacillus*–*Apilactobacillus* clades (Fig. 4). In the *Bombilactobacillus* clade, there was also a decrease in the number of genes assigned to category G (carbohydrate transport and metabolism) when compared to sister genus *Companilactobacillus*; however, this difference was not statistically significant. Lipid transport and metabolism (I) was also statistically significantly less represented in bee-associated clades (Fig. 4) (Kruskal–Wallis test).

## DISCUSSION

### Reductive evolution might be a hallmark of bee-associated microbes

One of the distinctive phenotypic traits found in FLAB is the preference for fructose over glucose (fructophily) (20). This unusual metabolic trait is hypothesized to have evolved in a background of lack of alcoholic fermentation and subsequent rewiring of sugar metabolism toward an alternative type of fermentation that involves fructose and mannitol as substrate and product, respectively (18, 20, 22, 24). We found that one of the most important genomic features for the random forest classifier was the absence of alcohol dehydrogenase (Adh) activity (total or partial absence of the encoding *adhE* gene), which is the main determinant for fructophily in FLAB (19). Although phenotypic information regarding fructophily is limited, by inspecting the available literature, we found that at least 50% of the bee-associated LAB that lack Adh activity have been described as fructophilic (Fig. S1) (14, 15, 18, 20, 21, 29). This suggests a link between loss of alcohol dehydrogenase function and adaptation to the bee environment in LAB. Interestingly, even in clades where loss of AdhE is pervasive, some species encode apparently functional AdhE enzymes. For instance, in the *Apilactobacillus–Fructilactobacillus* clade, some species totally lack AdhE (2); others only lack the Adh domain (10); and some have apparently functional enzymes (7). Since these three patterns do not strictly align with the phylogenetic relationships between these species, it seems plausible that loss of Adh activity happened multiple times in this niche, both in the most recent common ancestor (MRCA) of entire clades and in single species, and can happen in a stepwise manner.

While many other genes were lost in bee-associated bacterial species when compared to their counterparts that occupy distinct ecological niches, indicating that reductive evolution is pervasive in this ecological setting, specifically the loss of alcoholic fermentation-related genes seems to be strongly associated with other microorganisms thriving in the floral environment. This is supported not only by the multiple independent instances of loss of *adhE* across bee-associated LAB but also by the loss of the entire alcoholic fermentation pathway in a fructophilic floral yeast clade (*Wickerhamiella*/*Starmerella* clade, kingdom Fungi) (30, 31). Many of the species composing the *Wickerhamiella*/*Starmerella* yeast clade are intimately associated with bees and are many times isolated together with FLAB, from the same samples (32). Remarkably, the loss of alcoholic fermentation in this yeast clade is also seemingly associated with the emergence of fructophily, possibly also linked to a major metabolic rewiring toward the alternative mannitol fermentation pathway that uses fructose (and not glucose) as a substrate (33).

Similarly to FLAB, this yeast lineage also lost many metabolic abilities (31, 34, 35), supporting the notion that reduction of metabolic abilities might be pervasive across microorganisms adapting to the bee environment. Whether the set of genes and functions lost were similar among bee-associated yeasts and bacteria awaits further investigations.

### Were the losses adaptive or neutral byproducts of the switch to a new environment?

Gene loss is an important genetic mechanism contributing to phenotypic variation across the tree of life (34, 36–39). Gene losses can be adaptive in the sense that they are required for adaptation to a new environment, but they can also occur in a neutral fashion, as a consequence of relaxed selection following adaptation. If the time point in evolution when these losses occurred coincides with a period during which adaptation evolved, these two scenarios are difficult to distinguish without experimental validation. However, we can draw some inferences based on the putative functions of the identified lost genes. Using complementary approaches involving machine learning and evolutionary analysis of homolog family sizes across LAB, we found that most predicted lost functions are related with metabolism. More specifically, we inferred that some of these losses might have occurred concomitantly with the adaptation period, meaning that they can be either adaptive or neutral. Although experimental validation of the phenotypic consequences of inactivation of these genes in a distinct genetic background would be required to distinguish between these two hypotheses, their functions (mainly sugar, lipid, and amino acid metabolism) suggest that losses were most likely the result of relaxed selection.

Interestingly, we find not only the same functions but also the same genes were sometimes lost independently across bee-associated LAB. While the recurrent evolution of the same trait under similar selective pressures across distantly related species can reflect the power of selection, it can also occur for reasons other than selection on that trait. If sources of variation are biased and only allow a limited number of changes, distantly related species may easily evolve convergent traits by non-adaptive processes owing to relaxed selection (40), constraints (5), mutational biases (11), or shared genetic variation (12). Genetic constraints in particular (e.g., shared pleiotropic or epistatic effects) can be important drivers of patterns of convergence. For instance, if occupation of the bee-associated environment leads to the evolution of faster fructose consumption through loss of alcoholic fermentation, then other fermentation-related features may also evolve convergently as a consequence, although they are directly not under selection.

### The potential of statistical learning approaches for detecting fingerprints of adaptation

Machine learning algorithms generally make predictions about unknown data based on training and learning with known data. However, detecting the patterns in known data that make a certain feature of interest predictable can be extremely valuable to answer scientific questions, especially when those questions are better answered when analyzing voluminous amounts and distinct types of data. Machine learning is an emerging powerful approach that has been used, for instance, to uncover the molecular bases of certain metabolic functions (27, 28, 41, 42) or to find phenotypic and genomic signatures of convergent evolution of certain ecological traits in yeasts (26). Here we reinforce the power of this approach by uncovering common gene loss events associated with adaptation to the bee environment. We not only found genes that have been previously associated with FLAB bacteria and linked to particular phenotypes (*adhE*–fructophily), but new ones were also uncovered and shown to have been recurrently and independently lost across bee-associated LAB. For instance, absence of *glxK*/*garK*, encoding a glycerate 2-kinase, and *ohyA*, encoding an oleate dehydratase, have, to our knowledge, not been previously reported. *glxK/garK* is involved in the glyoxylate and glucarate/galactarate utilization pathways, and *ohyA* is possibly associated with the conversion of unsaturated fatty acids and might play a role in the evasion of the human host innate immune response by *Staphylococcus aureus*, through inactivation of antimicrobial unsaturated fatty acids (43).

We hypothesized that since bee-associated species largely stem from four clades, phylogenetic signal could be driving the machine learning classification. However, several pieces of evidence suggest that phylogenetic relatedness is not the only driver of this classification. First, we obtained accurate classifications across the entire phylogenetic spectrum both for multiple distantly related entire clades and for single species (i.e., *Holzapfelia floricola)* (Fig. 1). This suggests that, apart from the common features shared by species belonging to monophyletic clades, some of the features are also shared with distantly related single species that are clustered within non-bee-associated clades. Second, looking at the most important KEGG annotations for the RF classifier (Fig. 2B and C), we could observe that many are absent from more than 75% of the species, suggesting that the same losses happened in multiple distantly related clades, independently. This indicates that irrespective of the phylogenetic relatedness, the same featured independently evolved in, at least, more than one clade.

Overall, these results underscore how convergent evolutionary paths can be followed by microorganisms thriving in similar ecological contexts. Moreover, it underscores the power of machine learning to uncover fingerprints of convergent evolution, opening new and efficient avenues for the study of ecological adaptation.

## MATERIALS AND METHODS

### Genome annotation and phylogenetic inference

A total of 369 representative genomes of the family Lactobacillaceae and two outgroups (*Lactococcus lactis* and *Enterococcus massiliensis*) were retrieved from the National Center for Biotechnology Information (NCBI) (19 January 2023). For all species, the complete proteome was predicted with AUGUSTUS v.3.3.3 (44) using the complete gene model and *Staphylococcus aureus* as reference.

The species phylogeny was reconstructed with SCOs present in at least 50% of the species obtained using OrthoFinder v.2.5.4 (45) from the AUGUSTUS predicted proteomes. The SCOs were aligned independently using MAFFT v.7.407 (46) and then concatenated using a Python script (https://github.com/santiagosnchez/ConcatFasta). The concatenated aligned file composed of 180 SCOs was then used to infer a maximum likelihood phylogeny using IQ-TREE v.1.6.11 (47) with a partition flag (-spp), an automatic detection of the best-fitting model, and 1,000 ultrafast bootstrap replicates (48). The phylogeny was rooted using *Lactococcus lactis* and *Enterococcus massiliensis* (21).

### HMM-based search of *adhE* and *ohyA* across LAB

To search for *adhE* and *ohyA* across all strains, first, an HMM profile was constructed. For that, the reviewed protein of AdhE for *Escherichia coli* was recovered from UniProt (P0A9Q7), and OhyA for *Lactobacillus amylolyticus* (WP_127345688) was used as query for a BLASTp search in the NCBI refseq database. Hits with an *e* value lower than 0.001 were retrieved, up to a maximum of 100 hits. The sequences recovered were then aligned with MAFFT v.7.407 (46), and an HMM profile for each gene was constructed in HMMER v.3.3.3 (49). The HMM profile was used to score the presence/absence of each gene and the respective copy number using Orthofisher v.1.0.5 (25) with default parameters. Detected partial sequences of AdhE were subsequently analyzed through BLASTp on NCBI to assess which domain of the bifunctional protein was missing (aldehyde dehydrogenase or alcohol dehydrogenase).

### Inference of gene losses

Functional genomic annotations were performed using KofamScan (50) against the KEGG database (v.2022–12-31) with default settings. To minimize false-positive assignments, taxon-specific profile databases were used; in this case, prokaryotic proteins were annotated using “prokaryote.hal.” Proteins, for which the KofamScan did not yield a functional annotation (no K number assigned), were not reported in the output (“--no-report-unannotated”). Species for which a low number of proteins were mapped to a functional KEGG annotation were excluded from posterior analysis. To define the exclusion threshold, a ratio between the number of coding sequences and annotated KEGG was determined for each species. Species with a ratio above 3 were excluded (Fig. S2).

To infer losses across our data set, we used two approaches. The first implied using the tool COGclassifier (51). COGclassifier assigns prokaryote protein sequences into COG (Cluster of Orthologous Genes) functional categories. This analysis was performed for all strains in bee-associated clades and their respective sister clades. The proportion of proteins assigned to each category was calculated by dividing the number of annotated proteins from each strain by the total number of annotated proteins for a given category.

To infer gene losses in specific nodes, we used a parsimony-based approach implemented in Count (52). Losses were inferred under a Wagner parsimony method (53), which assumes an unknown ancestral state and allows both transitions from 0 (loss) to 1 (gain) and the reverse. KEGG functional annotations for all species and the species tree topology (Fig. 1) were used. The number of losses were inferred in the MRCA of the bee-associated clades and respective sister clades, and the KEGG Orthology (KO) annotations involved were retrieved and subsequently analyzed with KEGGmapper to determine their functional categories. In both cases, KEGG annotations generated by KofamScan were used as input.

### Machine learning

To test whether we could predict the floral-bee niche from KEGG Orthology genomic data, we used a random forest algorithm. We trained a machine learning algorithm built by an XGBoost v.1.7.3 random forest classifier (XGBRFClassifier()) (54) with the parameters “max_depth = 12” and “n_estimators = 100”; all other parameters were in their default settings. The max_depth parameter specifies the depth of each decision tree, determining how complex the random forest will be to prevent overfitting while maintaining accuracy. The n_estimators parameter specifies the number of decision trees in the forest. After testing the increase in accuracy while increasing each of these parameters, we found that having a higher max_depth or more decision trees per random forest did not further increase accuracy.

The random forest algorithm was trained on 90% of the data and used the remaining 10% for cross-validation, using the RepeatedStratifiedKFold and cross_val_score functions from the sklearn.model_selection v.1.2.1 package (55) . Cross validation is a method for assessing accuracy involving 10 trials, each of which holds back a random 10% of the training data for testing (54, 55) Given the unbalanced nature of the data set, we used balanced accuracy, which takes the mean of the true positive rate and the true negative rate, since there were unequal numbers of growers and non-growers in many of these substrates. For both measures, an accuracy value of 50% would be equivalent to randomly guessing. Top features were automatically generated by the XGBRFClassifier function using Gini importance, which uses node impurity (the amount of variance in environmental niche for strains that either have or do not have this KEGG Ortholog).

### Statistical analysis

All statistical analyses were performed in RStudio (R v.4.2.1). All data sets were first tested for normality with Shapiro–Wilk normality test (shapiro.test). Graphs and statistics were done with the package ggstatsplot (56), where for each case it was specified the type of analysis (parametric or non-parametric) according to Shapiro–Wilk results. For all statistical tests, “***” corresponds to a *P* value of <0.001; “**” corresponds to a *P* value of <0.01; and “*” corresponds to a *P* value of <0.05.

For phylogeny-informed statistical analyses, namely, differences in genomes size, protein-coding genes and GC content between different niches (bee-associated and other), a phylogenetic analysis of variance using the function “phylANOVA” implemented in the R package phytools (23, 57) was used.

To assess if there was a dependence relationship between niche (bee / other) and AdhE (presence / absence) when accounting for phylogeny, we conducted model testing of four hypothesis using phytools v 2.1.1 (57). Specifically, we tested if (i) the niche pattern and the occurrence of AdhE are independent of one another (null hypothesis); (ii) the niche pattern is dependent on AdhE; (iii) the pattern of occurrence of AdhE is dependent on niche; and (iv) the patterns of niche and AdhE are interdependent. We evaluated model fit using weighted Akaike information criterion and compared the best fitting model to the null hypothesis using a chi-squared test. We also did the same test for the OhyA protein, which has a similar distribution as AdhE. The hypotheses tested were the same.

## SUPPLEMENTAL MATERIAL

Fig. S1 aem.01257-24-s0001.pdf Maximum likelihood phylogenomic tree comprising 369 Lactobacillaceae species.

Fig. S2 aem.01257-24-s0002.pdf Ratio between the number of protein-coding genes and the number of annotated proteins to a KEGG number (KO).

Fig. S3 aem.01257-24-s0003.pdf Boxplots show the proportion of annotated proteins in COG categories.

Table S1 aem.01257-24-s0004.xlsx List of Lactobacillaceae species used in this work.

Table S2 aem.01257-24-s0005.xlsx Information about the machine learning raw results.

Table S3 aem.01257-24-s0006.xlsx List of KEGG numbers gained, lost, contracted, and expanded in the four main clades of bee-associated species, given by counts.

Table S4 aem.01257-24-s0007.xlsx List of the annotated proteins by COG category for the clades of bee-associated species.

ASM does not own the copyrights to Supplemental Material that may be linked to, or accessed through, an article. The authors have granted ASM a non-exclusive, world-wide license to publish the Supplemental Material files. Please contact the corresponding author directly for reuse.
