# Supplementary material for: Convergent reductive evolution in bee-associated lactic acid bacteria
Source: Appl Environ Microbiol. 2024 Oct 23;90(11):e01257-24. doi: 10.1128/aem.01257-24 (PMC11577768; doi:10.1128/aem.01257-24)
Supplement: Fig. S1 — Maximum likelihood phylogenomic tree comprising 369 Lactobacillaceae species. [file aem.01257-24-s0001.pdf]

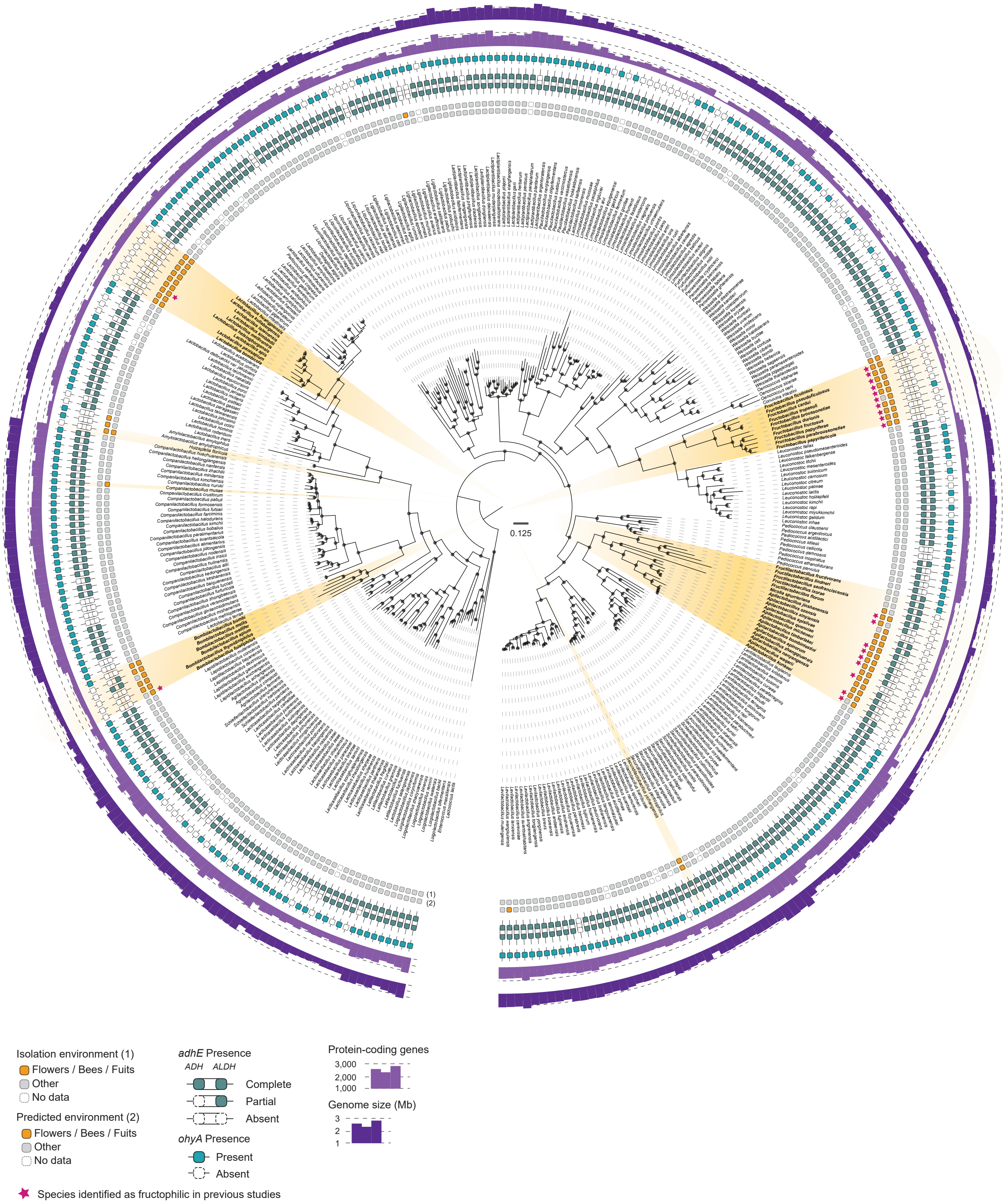

Figure S1 - Maximum likelihood phylogenomic tree comprising 369 Lactobacillaceae species inferred from the concatenated alignment of 180 SCO and rooted with *Lactococcus lactis* and *Enterococcus massiliensis*. The isolation source and predicted environment are depicted in the first two rings, respectively. The species isolated from bee-associated environments are highlighted in yellow. Presence/absence of *adhE* and *ohyA*, number of protein-coding genes and genome size are also shown.
