# Supplementary material for: Convergent reductive evolution in bee-associated lactic acid bacteria
Source: Appl Environ Microbiol. 2024 Oct 23;90(11):e01257-24. doi: 10.1128/aem.01257-24 (PMC11577768; doi:10.1128/aem.01257-24)
Supplement: Fig. S2 — Ratio between the number of protein-coding genes and the number of annotated proteins to a KEGG number (KO). [file aem.01257-24-s0002.pdf]

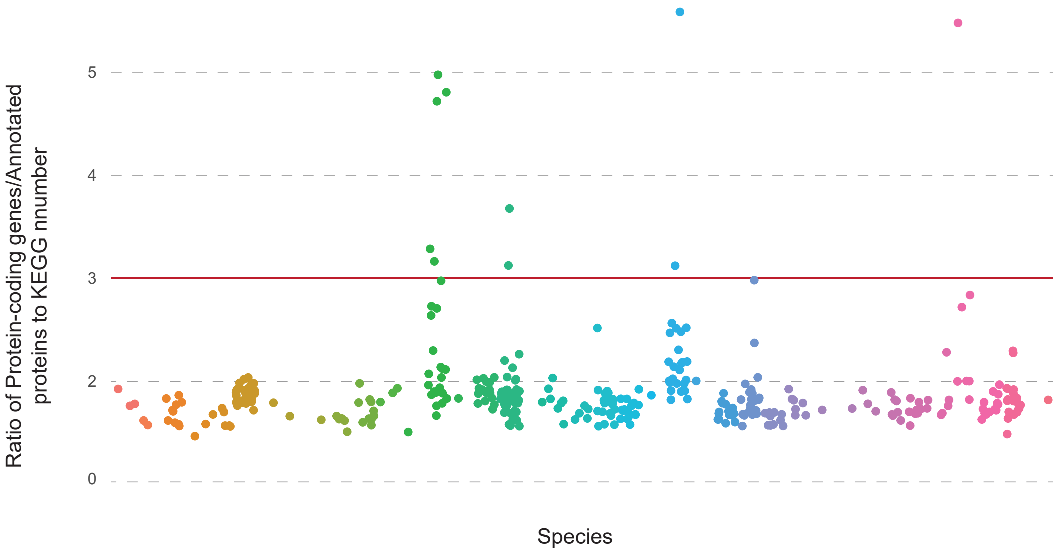

Figure S2 - Ratio between the number of protein-coding genes and the number of annotated proteins to a KEGG number (KO) for each species. The different genera are given by different colours. The threshold applied (ratio = 3) is marked by a red line.
