# Supplementary material for: Convergent reductive evolution in bee-associated lactic acid bacteria
Source: Appl Environ Microbiol. 2024 Oct 23;90(11):e01257-24. doi: 10.1128/aem.01257-24 (PMC11577768; doi:10.1128/aem.01257-24)
Supplement: Fig. S3 — Boxplots show the proportion of annotated proteins in COG categories. [file aem.01257-24-s0003.pdf]

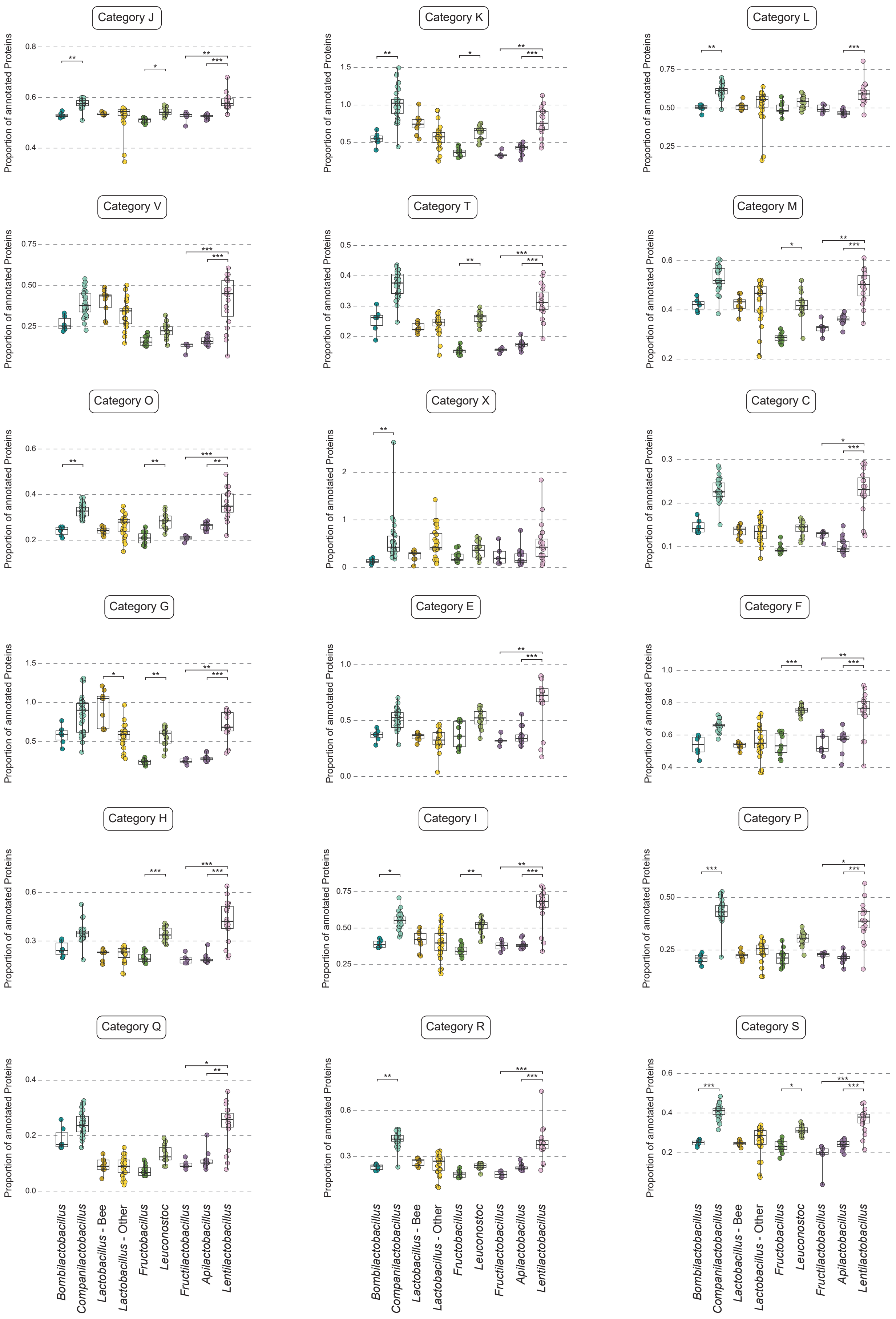

Figure S3 - Boxplots show the proportion of annotated proteins in COG categories for bee-associated and their respective sister clades. '***' corresponds to a p-value < 0.001, '**' p-value < 0.01, and '*' p-value < 0.05.
